# Valve-in-valve transcatheter pulmonary valve replacement in carcinoid heart disease: a case report

**DOI:** 10.1093/ehjcr/ytag220

**Published:** 2026-04-16

**Authors:** Giorgio Antonelli, Valeria Cammalleri, Annamaria Tavernese, Annunziata Nusca, Gian Paolo Ussia

**Affiliations:** Unit of Hemodynamics, Fondazione Policlinico Universitario Campus Bio-Medico, Via Alvaro del Portillo, 200, Roma 00128, Italy; Unit of Hemodynamics, Fondazione Policlinico Universitario Campus Bio-Medico, Via Alvaro del Portillo, 200, Roma 00128, Italy; Unit of Hemodynamics, Fondazione Policlinico Universitario Campus Bio-Medico, Via Alvaro del Portillo, 200, Roma 00128, Italy; Unit of Hemodynamics, Fondazione Policlinico Universitario Campus Bio-Medico, Via Alvaro del Portillo, 200, Roma 00128, Italy; Unit of Hemodynamics, Fondazione Policlinico Universitario Campus Bio-Medico, Via Alvaro del Portillo, 200, Roma 00128, Italy

**Keywords:** Case reports, Carcinoid heart disease, Transcatheter pulmonary valve replacement, Multimodality imaging

## Abstract

**Background:**

Carcinoid heart disease (CHD) commonly affects right-sided heart valves, often leading to progressive right heart failure (RHF) and structural valve deterioration.

**Case Summary:**

A 30-year-old male with CHD and prior bioprosthetic tricuspid and pulmonic valve replacements presented with New York Heart Association Class III symptoms and RHF. Imaging revealed significant degeneration of both valves. Given the high surgical risk (EuroSCORE II 9.12%), the Heart Team opted for transcatheter pulmonary valve replacement (TPVR). Despite challenging anatomy, a Melody valve (Medtronic) was successfully implanted, resulting in improved haemodynamics and no significant residual regurgitation.

**Discussion:**

Bioprosthetic failure in CHD poses significant treatment challenges. This case underscores the growing role of TPVR as a less invasive and effective alternative to surgery in high-risk patients, with advanced imaging playing a pivotal role in planning and execution.

Learning points

- Carcinoid heart disease (CHD) often leads to accelerated degeneration of right-sided bioprosthetic valves, resulting in right heart failure.
- Transcatheter pulmonary valve replacement (TPVR) offers a minimally invasive alternative to surgical reintervention for managing failing right-sided bioprostheses in CHD.
- Multimodality imaging is critical for procedural planning and ensuring safe valve deployment.

## Introduction

Carcinoid heart disease (CHD) is a rare but severe complication of metastatic neuroendocrine tumours (NETs), most commonly originating from the gastrointestinal tract. Chronic exposure to circulating vasoactive substances, particularly serotonin, leads to fibrotic plaque deposition on the endocardium and progressive dysfunction of right-sided cardiac valves.^1,2^

Surgical valve replacement is the standard treatment for symptomatic severe valvular disease in CHD; however, operative mortality remains high due to advanced systemic disease, hepatic dysfunction, and poor functional status.^3–5^

In high-risk patients with degenerated pulmonary bioprostheses, transcatheter pulmonary valve replacement (TPVR) offers a minimally invasive alternative to redo surgery.^6,7^

We report a case of valve-in-valve TPVR in a young patient with persistent NET activity and severe degeneration of a surgical pulmonary bioprosthesis, successfully treated using a dual-wire strategy.

## Summary figure

**Figure:**
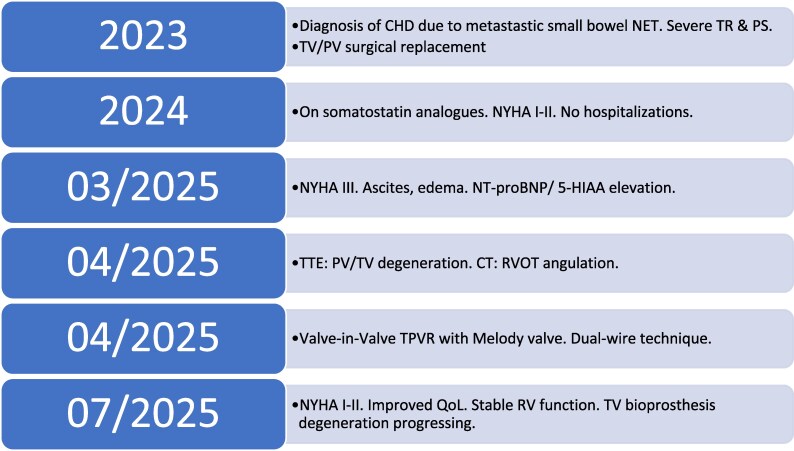


## Case presentation

A 30-year-old man with known CHD secondary to metastatic small bowel NET was referred for cardiology evaluation because of progressively worsening dyspnoea on mild exertion New York Heart Association (NYHA) functional class III.

On physical examination, he was haemodynamically stable with a blood pressure of 110/70 mmHg, heart rate of 82 bpm in sinus rhythm, and oxygen saturation of 97% on room air. Clinical signs of right heart failure were present, including hepatomegaly, moderate ascites, and bilateral mid-shin pitting oedema.

## Past medical history

Two years earlier, he had been diagnosed with metastatic small bowel NET complicated by carcinoid syndrome and treated with long-acting somatostatin analogues. CHD was subsequently diagnosed based on echocardiographic findings of severe tricuspid regurgitation and pulmonic stenosis. He underwent surgical valve replacement with Carpentier–Edwards bioprostheses (27-mm tricuspid and 23-mm pulmonic) (Edwards Lifesciences), with initial symptomatic improvement.

He had no other significant comorbidities and denied smoking or illicit drug use.

## Investigations

**Figure 1:**
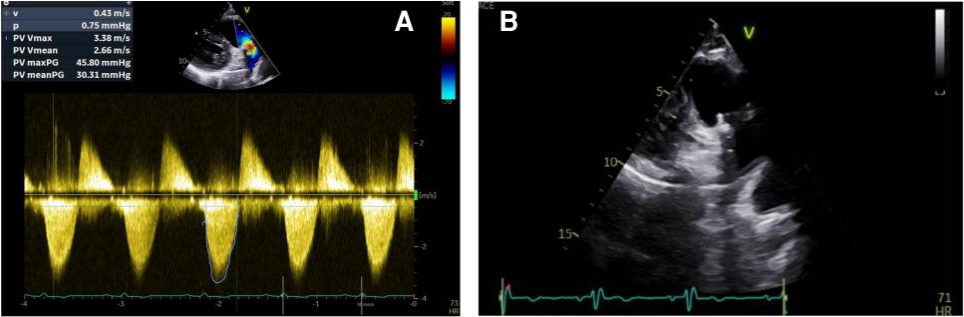
(*A*) parasternal short axis of the CW Doppler of pulmonary bioprosthesis. (*B*) Parasternal long axis right ventricular (RV) inflow showing the pulmonary bioprosthesis.

**Figure 2:**
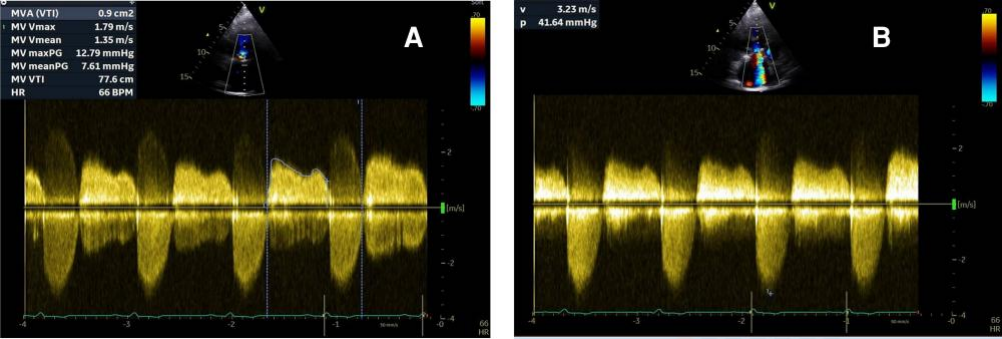
(*A*) CW Doppler apical 4-chamber (A4C) of tricuspid bioprosthesis. (*B*) CW Doppler apical 4-chamber of tricuspid regurgitation peak velocity (*V*_max_).

Transthoracic echocardiogram (TTE) revealed severe structural deterioration of both bioprosthetic valves. The pulmonic valve demonstrated a maximum gradient of 46 mmHg, a mean gradient of 30 mmHg, and a peak velocity (*V*_max_) of 3.4 m/s, with a reduced effective orifice area of 0.9 cm^2^ (see Supplementary material online, *Video S1*, *Figure 1A* and *B*). Pressure half-time was shortened to 87 ms. In addition, the tricuspid bioprosthesis showed a mean gradient of 8 mmHg (*Figure 2A*) with significant regurgitation (*Figure 2B*). Both bioprosthetic leaflets appeared thickened and exhibited restricted motion both is systole and diastole, consistent with degeneration.

Left ventricular ejection fraction was preserved (50–55%). Interventricular septal flattening produced a D-shaped left ventricle (eccentricity index 1.09), consistent with right ventricular pressure overload (see Supplementary material online, *Video S2*). The right ventricle was normal in size with preserved systolic function (tricuspid annular plane systolic excursion 19 mm, S’ wave velocity 17 cm/s, and fractional area change 41%), while the right atrium was dilated.

Electrocardiogram showed regular sinus rhythm, without significant abnormalities. Chest X-ray showed mild pleural effusion.

**Figure 3:**
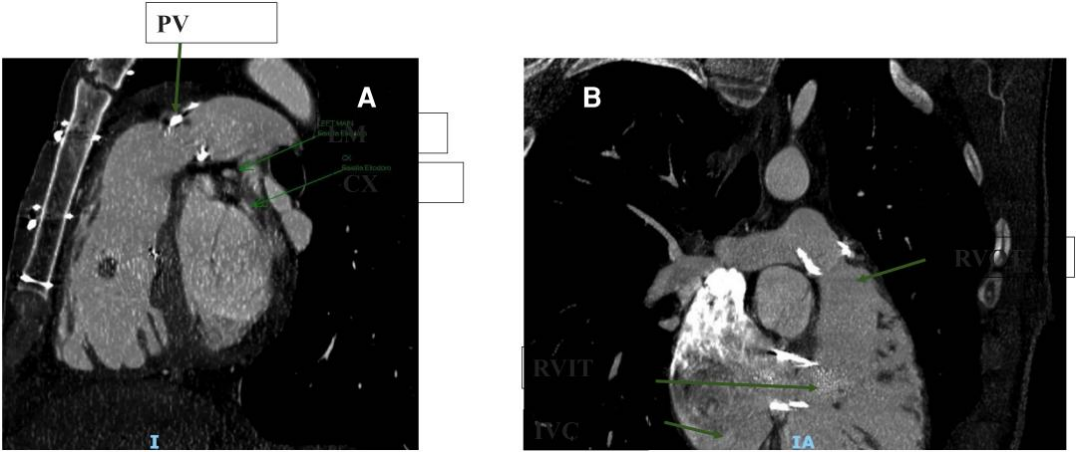
(*A*) CT (computed tomography) showing the distance between the pulmonary bioprosthesis and the coronary arteries. (*B*) CT showing the relationship between right ventricular inflow and outflow tracts. CX = circumflex artery; IVC = inferior vena cava; LM = left main; PV = pulmonary valve; RVIT = right ventricular inflow tract; RVOT = right ventricular outflow tract.

Cardiac computed tomography (CT) was performed for procedural planning and confirmed an adequate landing zone for TPVR with safe coronary clearance (*Figure 3A*) but demonstrated a challenging angulation between the inferior vena cava and the right ventricular inflow–outflow axis (*Figure 3B*).

Laboratory testing showed elevated Serum N-terminal pro–B-type natriuretic peptide (NT-proBNP) (950 pg/ml) and increased urinary 5-hydroxyindoleacetic acid (5-HIAA) (25 mg/24 h), confirming persistent NET activity despite somatostatin therapy.

Right heart catheterization revealed severe right-sided haemodynamic compromise. Right atrial pressure was 20 mmHg with prominent v-waves. Right ventricular pressure was 75/7 mmHg. Pulmonary artery pressure was 40/20 mmHg with a mean pressure of 30 mmHg. Pulmonary capillary wedge pressure was 16 mmHg. The transpulmonary gradient was 14 mmHg. Cardiac index was reduced to 1.54 L/min/m^2^. Pulmonary vascular resistance was 4 Wood units, and mixed venous oxygen saturation was around 64%.

## Management

The multidisciplinary Heart Team deemed the patient at high risk for redo surgery (EuroSCORE II 9.12%) due to advanced CHD, previous valve surgery, and clinical right heart failure. Oncologists actively participated in the decision-making process and recommended continuation of somatostatin analogue therapy, with potential escalation to interferon alfa if needed,^5^ and estimated a life expectancy exceeding 12 months assuming successful cardiac intervention.

After optimization of volume status, valve-in-valve TPVR was selected to improve symptoms, restore right ventricular haemodynamics, and allow continuation of oncologic therapy.

The procedure was performed under conscious sedation via right femoral venous access. Initial crossing of the tricuspid and pulmonic bioprostheses was achieved using a pigtail catheter and J-wire. Due to the pronounced angulation between the right ventricular inflow and outflow tracts (RVIT/RVOT), a 10-Fr Oscor Destino (Oscor) support catheter was required.

Pulmonary angiography confirmed severe pulmonary bioprosthetic dysfunction (see Supplementary material online, *Video S3*). An extra-stiff Lunderquist (Cook Medical) wire was advanced into the left pulmonary artery. Attempts to advance a 26-Fr DrySeal Flex (Gore) introducer sheath were unsuccessful due to instability related to RVOT angulation, even though it reached the pulmonary valve, it remained unstable at that level, prompting a change in procedural strategy, owing to angulation between the inferior vena cava and the RVOT (see Supplementary material online, *Video S4*).

A second parallel Lunderquist wire was therefore introduced to provide additional support, enabling successful sheath advancement using a dual-wire strategy (see Supplementary material online, *Video S5*).

**Figure 4:**
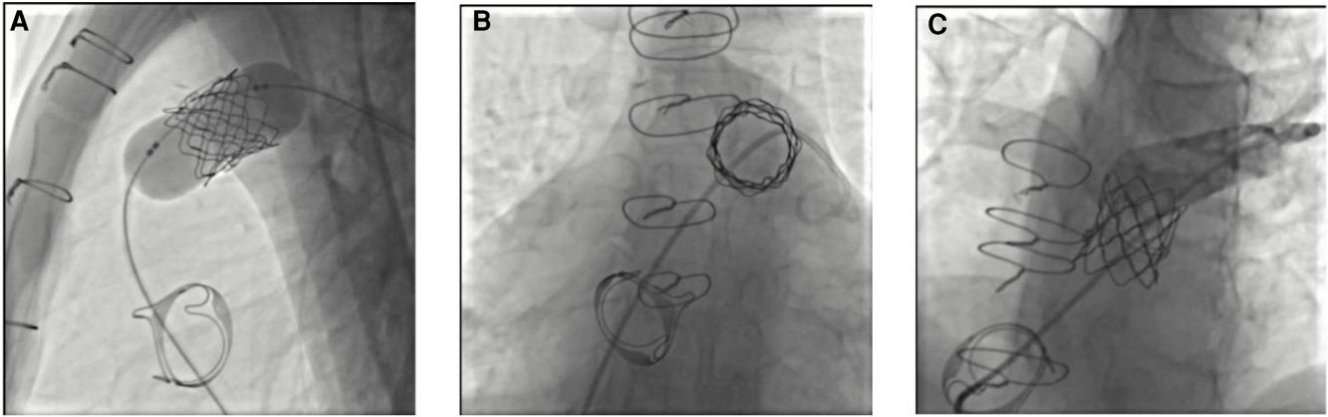
(*A*) fluoroscopic image of the deployment of the melody (medtronic) valve within the degenerated bioprosthesis. (*B*) Fluoroscopic image of the Melody (Medtronic) valve within the degenerated bioprosthesis. (*C*) Final angiography demonstrated excellent valve positioning and absence of paravalvular leak.

A 22-mm Melody valve (Medtronic) was deployed within the degenerated pulmonic bioprosthesis under angiographic guidance (see Supplementary material online, *Video S6*, *Figure 4A*). Final angiography confirmed optimal valve position without paravalvular leak (see Supplementary material online, *Video S7*, *Figure 4B* and *C*). Venous haemostasis was achieved using a Perclose ProGlide closure device (Abbott).

## Outcome and follow-up

**Figure 5:**
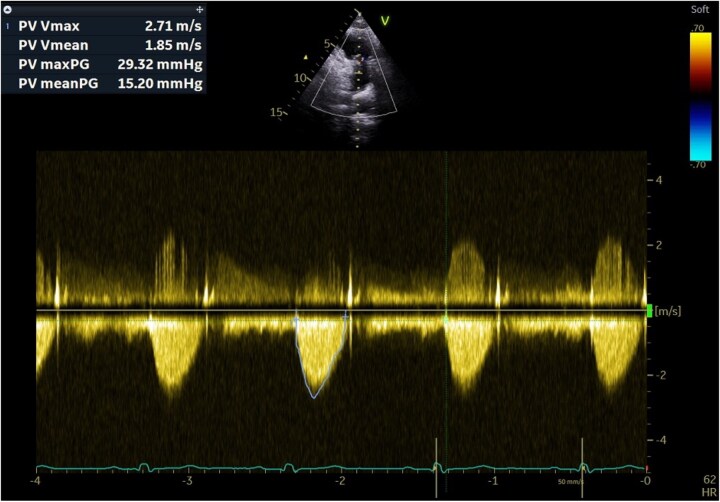
CW Doppler of the pulmonary valve after the procedure.

The post-procedural course was uncomplicated. TTE showed trivial residual pulmonary regurgitation and a reduced mean gradient of 14 mmHg. (*Figure 5*).

The patient was discharged on day three on vitamin K antagonist (VKA) therapy and continued somatostatin analogue treatment, as well as furosemide and empaglifozin, a sodium-glucose Co-transporter 2 inhibitor (SGLT2I).

At 3-month follow-up, he reported marked symptomatic improvement (NYHA I–II). Repeat TTE showed sustained prosthetic valve function (see Supplementary material online, *Video S8*). stable right ventricular size and function, and reduced right atrial dimensions (see Supplementary material online, *Video S9*). However, degeneration of the tricuspid bioprosthesis has further progressed (see Supplementary material online, *Video S10*).

## Discussion

CHD is a rare but severe complication of metastatic NETs, characterized by fibrotic involvement of right-sided cardiac valves.^4^ The tricuspid and pulmonic valves are most commonly affected, leading to regurgitation, stenosis, or mixed lesions and progressive right heart failure.^5^ Although surgical valve replacement remains the standard treatment for symptomatic severe valvular disease, perioperative mortality remains high (18%–20%),^3^ reflecting carcinoid-related systemic illness, hepatic dysfunction, and metastatic disease burden.^8^

At first, bioprosthetic valves are generally preferred over mechanical prostheses in CHD due to thrombosis risk in the low-pressure right-sided circulation, bleeding risk associated with lifelong anticoagulation, and the feasibility of future transcatheter valve-in-valve interventions.^5^ However, ongoing exposure to vasoactive and fibrogenic mediators results in accelerated bioprosthetic degeneration,^9–11^ often necessitating repeat intervention, for which redo surgery carries substantial risk

Valve-in-valve TPVR has emerged as a minimally invasive alternative for selected high-risk patients and is increasingly applied to structural valve degeneration in CHD.^7^

Our institutional experience supports TPVR as a reduced-risk procedure capable of improving symptoms and quality of life over short-term follow-up.^12^

In the present case, the pulmonic bioprosthesis was treated first to reduce right ventricular pressure overload and promote reverse remodelling before potential valve-in-valve intervention on the tricuspid bioprosthesis.

This case highlights the importance of meticulous pre-procedural planning, particularly with cardiac CT, to assess right heart and coronary anatomy,^5^ as well as the value of multidisciplinary Heart Team collaboration. Advanced catheter techniques, such as dual-wire support, may be required to overcome challenging anatomy and ensure stable device delivery, especially in pulmonary valve interventions where limited calcification necessitates precise landing-zone preparation.^12^

The Melody valve, constructed from bovine jugular vein tissue rather than pericardium, was selected based on operator familiarity and its potential resistance to serotonin-mediated fibrosis, as suggested by our prior experience in another patient with a 2-year follow-up.^12^

## Conclusions

Valve-in-valve TPVR is a safe and effective alternative to redo surgery in patients with CHD and failing pulmonary bioprostheses who are at high surgical risk. Multimodality imaging, multidisciplinary planning, and advanced catheter techniques are essential to optimize outcomes and improve quality of life.

## Supplementary Material

ytag220_Supplementary_Data

## Data Availability

The data underlying this article will be shared on reasonable request to the corresponding author.

## Lead author biography

Dr. Giorgio Antonelli is a young cardiologist and PhD candidate at Campus Bio-Medico University. His primary areas of interest are valvular heart disease and interventional and structural cardiology, with a focus on advancing minimally invasive techniques and improving patient outcomes

## Notes

**Consent:** The authors confirm that written consent for submission and publication including images and texts has been obtained from the patient in line the Committee on Publication Ethics guidance and under the review board of the hospital administration.
